# Impact of dietary protein on lipid metabolism-related gene expression in porcine adipose tissue

**DOI:** 10.1186/1743-7075-7-6

**Published:** 2010-01-21

**Authors:** Sumei Zhao, Jing Wang, Xinlei Song, Xi Zhang, Changrong Ge, Shizheng Gao

**Affiliations:** 1 Yunnan Key Laboratory of Animal Nutrition and Feed Science, Yunnan Agricultural University, Kunming 650201, China

## Abstract

**Background:**

High dietary protein can reduce fat deposition in animal subcutaneous adipose tissue, but little is known about the mechanism.

**Methods:**

Sixty Wujin pigs of about 15 kg weight were fed either high protein (HP: 18%) or low protein (LP: 14%) diets, and slaughtered at body weights of 30, 60 or 100 kg. Bloods were collected to measure serum parameters. Subcutaneous adipose tissues were sampled for determination of adipocyte size, protein content, lipid metabolism-related gene expression, and enzyme activities.

**Results:**

HP significantly reduced adipocyte size, fat meat percentage and backfat thickness, but significantly increased daily gain, lean meat percentage and loin eye area at 60 and 100 kg. Serum free fatty acid and triglyceride concentrations in the HP group were significantly higher than in the LP group. Serum glucose and insulin concentrations were not significantly affected by dietary protein at any body weight. HP significantly reduced gene expression of acetyl CoA carboxylase (ACC), fatty acid synthase (FAS) and sterol regulatory element binding protein 1c (SREBP-1c) at 60 kg and 100 kg; however, the mRNA level and enzyme activity of FAS were increased at 30 kg. HP promoted gene and protein expression and enzyme activities of lipoprotein lipase (LPL), carmitine palmtoyltransferase-1B (CPT-1B), peroxisome proliferator-activated receptor *γ *(PPAR*γ*) and adipocyte-fatty acid binding proteins (A-FABP) at 60 kg, but reduced their expression at 100 kg.

Gene expression and enzyme activity of hormone sensitive lipase (HSL) was reduced markedly at 60 kg but increased at 100 kg by the high dietary protein. Levels of mRNA, enzyme activities and protein expression of ACC, FAS, SREBP-1c and PPAR*γ *in both LP and HP groups increased with increasing body weight. However, gene and protein expression levels/enzyme activities of LPL, CPT-1B, A-FABP and HSL in both groups were higher at 60 kg than at 30 and 100 kg.

**Conclusion:**

Fat deposition in Wujin pigs fed high dietary protein for 25 weeks was reduced mainly by depression of lipogenic gene expression. The mechanism of lipid transport, lipolysis and oxidation in adipose tissue regulated by dietary protein appeared to be different at 60 kg and 100 kg body weights.

## Background

The deposition of fat in meat animals may be beneficial or undesirable depending on its quantity and location. Excessive deposition of fat, except intramuscular fat, has been recognized as detrimental to carcass quality, and constitutes a health risk to human consumers [1]. The synthesis of subcutaneous adipose tissue triglycerides - the major constituents of depot fat - either proceeds from fatty acids synthesized *de novo *in that tissue [2,3] or from fatty acids obtained from circulating triglycerides as a result of adipose tissue lipoprotein lipase activity [4]. This suggests that the relative rates between synthesis and degradation of fatty acids determining deposition or utilization of depot fat in subcutaneous adipose tissue may be indicative of total body fat in animals and obesity in humans.

Processes that determine fat deposition in adipose tissue include the rates of fat uptake, *de novo *fatty acid synthesis, triacylglycerol synthesis, lipid degradation and transport processes of fatty acids [5]. Several key factors are involved in lipid metabolism in adipose tissues. Lipogenic enzymes include acetyl-CoA carboxylase (ACC), fatty acid synthase (FAS), malic enzyme (ME) and glucose-6-phosphate dehydrogenase (G-6-PDH), and changes in their activities can alter the rates of biosynthesis of fatty acids [6-11]. Moreover, hormone-sensitive lipase (HSL) and carnitine palmitoyltransferase 1(CPT-1) are rate limiting enzymes in catabolism, responsible for hydrolysis of triglycerides and importing esters of fatty acids to mitochondria for *β*-oxidation [12,13]. Lipoprotein lipase (LPL) is the rate limiting enzyme for the conversion of chylomicrons and VLDL into chylomicron remnants and LDL. LPL is regarded as rate limiting for the transfer of fatty acids into tissues [12]. Furthermore, sterol regulatory element binding protein 1(SREBP-1), peroxisome proliferator-activated receptor *γ *(PPAR*γ*) and adipose fatty acids-binding protein (A-FABP, also known as FABP4), have recently been identified as being implicated in lipid metabolism in adipose tissue by regulating gene expression of enzymes or proteins involved in lipid metabolism, or by transporting fatty acids [14-18].

The Wujin pig is a typical local breed in Yunnan province, China. Compared with other breeds, the Wujin pig has higher body fat, especially of intramuscular fat [19]. Because of the similarity of adipose tissue lipid metabolism in pigs and humans, the fatty pig is an ideal model for investigation of lipid metabolism in human adipose tissue [20,21].

Previous reports have revealed that high dietary protein can reduce carcass fat contents of pigs [22-26]. Little is known about the regulatory mechanisms of dietary protein on porcine adipose tissue lipid metabolism, and of the effect of dietary protein levels on gene expression relating to lipid metabolism. This study attempts to clarify some aspects of these, using Wujin pigs as an experimental model.

## Methods

All experimental procedures were approved by the Yunnan Agricultural University Committee of Laboratory Animal Care.

### Experimental diets

Dietary protein was formulated at two levels: low (14%, LP) and high (18%, HP), these being 2% below and above the Chinese feeding standard for local breed pigs [27]. Digestive energy of two groups was kept the same (13.56 MJ/kg). Composition and calculated nutrient contents of the experimental diets are presented in table 1.

**Table 1:** Composition of diets (%, as fed basis)

	LP^1^	HP^2^
Corn	73.72	60.46
Wheat bran	10.00	15.00
Soybean meal	8.38	16.14
Fish meal	4.50	5.00
Soybean oil	1.00	1.00
Monocalcium phosphate	0.20	0.20
Limestone	0.90	0.90
Salt	0.30	0.30
Premix^3^	1.00	1.00
Calculated nutritional composition^4^		
Crude protein	14.00	18.00
Total lysine	0.96	0.96
Digestive energy (MJ/kg)	13.56	13.56
Calcium	0.64	0.64
Total phosphorus	0.55	0.55
Available phosphorus	0.29	0.29

^1 ^LP: low protein diet.

^2 ^HP: high protein diet

^3^Vitamin premix provided the following per kilogram of diet: vitamin A, 8,267 IU; vitamin D2, 2,480 IU; vitamin E, 66 IU; menadionine [as menadionine pyrimidinol bisulfite complex], 6.2 mg; riboflavin, 10 mg; Ca d-pantothenic acid, 37 mg; niacin, 66 mg; vitamin B12, 45 *μ*g; d-biotin, 331 *μ*g; folic acid, 2.5 mg; pyridoxine, 3.31 mg; thiamine, 3.31 mg; vitamin C, 83 *μ*g; Trace mineral premix provided the following per kilogram of diet: Zn, 127 mg; Fe, 127 mg; Mn, 20 mg; Cu, 12.7 mg; I0.80 mg, as zinc sulfate, ferrous sulfate, manganese sulfate, copper sulfate, ethylenediamine dihydriodide, respectively, with calcium carbonate as the carrier, providing 0.3 mg Se per kilogram of diet.

^4 ^Nutritional compositions were calculated values based on China Feed Database.

### Pigs and housing

Two males and two females, weight approximately 15 kg, were selected from each of 15 litters. One male and one female constituted a unit, giving a total of 30 units, which were randomly divided into two main groups according to their diet (LP or HP). The pigs were individually housed in an insulated but unheated shed with a half slatted floor at the research station of Yunnan Agricultural University (March to October). They were given free access to water from nipple drinkers and their diets were provided *ad libitum*. They were weighed individually for calculation of average daily gain.

At body weights of 30 kg (end of the grower period), 60 kg (end of the early-finishing period) and 100 kg (end of the late-finishing period), groups of pigs were transported to the Yunnan Agricultural Center Meats Laboratory, and were slaughtered by exsanguination after electrical stunning. Blood samples were collected. Sera were separated by low speed centrifugation and stored at -20°C until required. Subcutaneous adipose tissue samples were taken from between the second and fourth thoracic vertebrae, snap frozen in liquid nitrogen, and stored at -80°C prior to analysis.

### Determination of adipocyte size

Adipocytes were obtained by collagenase treatment of fresh tissue from each sample. Adipocyte size was determined according to the method of Etherton and Chung [28]. Briefly, aliquots (10^3 ^cells) of isolated adipocytes were digitally photographed using a photomicroscopy system, and individual diameters (*μ*m) were measured using an image analysis system (Optimas 6.5; Media Cybernetics, Silver Spring, MD).

### Serum analysis

Serum free fatty acids (FFA), triglycerides (TG) were determined with commercial kits (Nanjing Jiancheng Biochemical Reagent Co., Nanjing, China).

Serum insulin concentrations were measured with a commercial multi-species RIA kit (Beijing North Institute of Biotechnology) that had been validated for measuring porcine serum samples. Detection limits for insulin were 0.2 ng/mL (1.0 *μ*IU/mL) with intra- and inter-assay coefficients of variation of 5% and 10%, respectively.

### Adipose tissue enzyme activity analyses

The activities of acetyl-CoA carboxylase (ACC, EC 6.4.1.2), fatty acid synthase (FAS, EC 2.3.1.85), malic enzyme (ME, EC 1.1.1.40), glucose-6-phosphate dehydrogenase (G-6-PDH, EC 1.1.1.49), hormone-sensitive lipase (HSL, EC 3.1.1.3) and lipoprotein lipase (LPL, EC 3.1.1.34) were determined in subcutaneous adipose tissue homogenates. Approximately 1 g adipose tissue was homogenized in 3 mL 10 mM HEPES buffer containing 0.25 M sucrose, 1 mM EDTA, and 1 mM dithiothreitol, and then centrifuged at 100,000 *g *for 30 min at 4°C. Supernatants were collected and used for the enzyme assays. The activity of ACC was assayed by the H^14^CO_3_-fixation method [29,30]. Spectrophotometric assays were used for ME [31], G-6-PDH [32], and FAS [33,34]. LPL activity was determined using a commercial kit (Nanjing Jiancheng Biochemical Reagent Co) according to the manufacturer's instructions. The assay for HSL activity was based on a cholesterol esterase assay described by Osterlund et al. [35]. The CPT-1 activity was assayed directly using whole mitochondrial isolates which were resuspended at 0°C in 0.3 M sucrose containing 10 mM Tris-HCl, pH 7.4, and 1 mM EGTA, sonicated, then centrifuged at 20,000 g for 40 min at 4°C to obtain a mitochondrial supernatant [36]. All assays were conducted in the range of linearity with respect to amount of enzyme and time. Soluble protein in the tissue supernatants was measured according to the method of Bradford [37], using bovine serum albumin as the standard. Unless otherwise noted, all reagents were obtained from Sigma (USA).

### RNA extraction

Total RNA was extracted from subcutaneous adipose tissue using TRIZOL reagent (Invitrogen, USA) according to the manufacturer's protocol, and quantified by measurement of optical density at 260 nm. Ratios of absorption (260/280 nm) of all preparations were between 1.6 and 1.8. Aliquots of RNA samples were subjected to electrophoresis in a 1% ethidium bromide-stained 1.4% agarose formaldehyde gel to verify their integrity.

### Real-Time quantitative RT-PCR

Reverse transcription was performed using the RNA (2 *μ*g) in a final volume of 25 *μ*L containing 10 units MMLV reverse transcriptase (Promega, USA), 1 mM dNTP mixture (Promega, USA), 40 units recombinant RNasin ribonuclease inhibitor (Promega, USA) and 0.5 *μ*g oligo (dT) 18 (Promega, USA) in sterile water and buffer supplied by the manufacturer. An aliquot of cDNA samples was mixed with 10 *μ*L iQ™ SYBR^® ^Green Supermix (Takala, Japan), in the presence of 0.5 *μ*M of each forward and reverse primer for porcine ACC, FAS, SREBP-1, LPL, HSL, CPT-1, and PPAR*γ*, A-FABP, then subjected to PCR under standard conditions (40 cycles). The specific primers used for real-time quantitative PCR are listed in table 2. As an internal control, the same reverse transcription products were also subjected to PCR in the presence of a second pair of primers specific to pig 18S rRNA. Mixtures were incubated in an iCyler iQ Real-time Detection System (Bio-Rad, USA) programmed to perform in 25 *μ*L of total reaction volume as per manufacturer's instructions. PCR conditions consisted of an initial denaturation step at 94°C for 4 min, then 40 cycles of denaturation/primer annealing/elongation (94°C for 50 s/62°C for 1 min/72°C for 1 min, respectively) with a final extension at 72°C for 10 min.

**Table 2:** Specific primers used for real-time quantitative PCR

Gene name^1^	Sequence	Ta	Product size (bp)	Genbank accession no.
18S rRNA	F: 5'-GCGGCTTTGGTGACTCTA-3' R:5'-CTGCCTCCTTGGATGTG-3'	60°C	194 [238-431]	NR_002170.3
ACC	F: 5'-ATG TTT CGG CAG TCC CTG AT-3' R: 5'-TGT GGA CCA GCT GAC CTT GA-3'	60°C	133 [4810-4942]	EF618729
FAS	F: 5'-AGC CTA ACT CCT CGC TGC AAT-3' R: 5'-TCC TTG GAA CCG TCT GTG TTC-3'	58°C	196 bp [504-699]	AY183428
SREBP-1	F: 5'-GCG ACG GTG CCT CTG GTA GT-3' R: 5'-CGC AAG ACG GCG GAT TTA-3'	62°C	218 bp [194-411]	AF102873
LPL	F: 5'-AAC TTG TGG CTG CCC TAT-3' R: 5'-GAC CCT CTG GTG AAT GTG-3'	55°C	367 bp [453-819]	X62984
HSL	F: 5'-GCT CCC ATC GTC AAG AAT C-3' R: 5'-TAA AGC GAA TGC GGT CC-3'	55°C	262 bp [2407-2668]	AJ000482
PPAR-*γ*	F: 5'-GAT TTC TCC AGC ATT TCC A-3' R: 5'-GCT CTT CGT GAG GTT TGT T-3'	60°C	184 bp [270-453]	DQ437884
CPT-1	F: 5'-ATG GTG GGC GAC TAA CT-3' R: 5'-TGC CTG CTG TCT GTG AG-3'	62°C	321 bp [681-1001]	AY181062
A-FABP	F: 5'-CAG GAA AGT CAA GAG CAC CA-3' R: 5'-TCG GGA CAA TAC ATC CAA CA-3'	55°C	227 bp [234-460]	AJ416020

^1 ^All porcine gene sequences.

Melting curve analyses were performed on all real time PCR reactions to confirm specificity and identity of the real time PCR products. Specificity was further confirmed by agarose gel electrophoresis. To confirm the absence of significant contamination in the total RNA preparation, cDNA was synthesized and control reactions were performed in the absence of reverse transcriptase. In all cases, no further PCR bands were detected.

The results (fold changes) were expressed as 2^-ΔΔCt ^with ΔΔCt = (Ct ij-Ct *β*-18S rRNA j)-(Ct il-Ct *β*-18S rRNA l), where Ct ij and Ct 18S rRNA j are the Ct for gene i and for 18S rRNA in a pool or a sample (named j) and where Ct i1 and Ct 18S rRNA 1 are the Ct in pool 1 or sample l, expressed as the standard. All primers used were designed by Primes Premier 5 and synthesized by Shanghai Shanggong Biological Company (Shanghai, China).

### Western blotting

Whole frozen adipose samples (0.5 g) were homogenized on ice in 700 *μ*L buffer A [50 mM Tris-HCl (pH 7.5), 50 mM NaF, 5 mM sodium pyrophosphate, 1 mM EDTA, 1 mM DTT, 0.1 mM phenylmethylsulfonyl fluoride, 10% glycerol] containing 1% Triton X-100, 5 *μ*M aprotinin, leupeptin and pepstatin. The lysates were centrifuged at 6,000 g for 20 min at 4°C to remove insoluble material. Thereafter, the supernatant extracts were collected and protein concentration determined using the method of Bradford [37]. The extracts were frozen at -80°C until western blot analyses were performed.

FABP, SREBP-1 and PPAR*γ *protein expression were measured according to the method described by Doran et al. [38]. Briefly, 50 *μ*g total whole cell protein extracts were electrophoresed on SDS-PAGE (12% resolving gel), transferred to a nitrocellulose membrane and probed overnight with a rabbit polyclonal anti-FABP, SREBP-1 and PPAR*γ *antibody (Sigma, USA; 1:500, 1:1,000 and 1:2,000 dilutions, respectively). The membranes were then probed with a goat anti-rabbit IgG-horseradish peroxidase conjugate (Sigma, USA; 1:20,000) for 1 h at room temperature. Blots were developed using the SuperSignal West Pico Chemiluminescent Substrate system (Bio-Rad, USA) and imaged on microfilm for image analysis and densitometry. Signal intensity was quantified using Quantity One 1-D analysis software (Bio-Rad, USA).

### Statistical analyses

The effects of dietary protein and body weight on serum parameters, adipocyte diameter, enzyme activities, and expression levels of lipid metabolism-related genes and protein were analyzed using the following model:

where *μ *is a population parameter. *α*_*i *_is effect of dietary protein, *β*_*j *_is effect of body weight, and *ε*_*ijl *_is random error or residual effects. The results were expressed as mean ± SE and differences were considered significant when *P *< 0.05 tested by 2-way analysis of variance (ANOVA) with Statistical Packages for SAS 9.0.

## Results

### Effect of dietary protein level on growth performance

The effects of dietary protein level on growth performance in Wujin pigs are shown in table 3. There were no significant differences in daily feed intake between the LP and HP groups during growth periods 15-30, 30-60 and 60-100 kg, but daily feed intake increased from 15 kg to 100 kg (P < 0.05). The daily gain in the HP group was significantly higher than in the LP group during the whole growth-finishing stages. The daily gain lessened with increasing body weight (P < 0.05). The effects of dietary protein levels on the gain: feed ratio was significant during the 15-30 and 30-60 kg periods.

**Table 3:** Effect of dietary protein on growth performance^1^

		15-30 kg	30-60 kg	60-100 kg
Daily feed intake (kg/d)	LP^2^	1.12 ± 0.01^a^	1.80 ± 0.06^b^	2.27 ± 0.08^c^
	HP^3^	1.13 ± 0.04^A^	1.82 ± 0.02^B^	2.24 ± 0.02^C^
Daily gain (g/d)	LP	392.11 ± 25.02^a^	500.56 ± 24.98^b^	461.49 ± 22.52^c^
	HP	470.62 ± 23.68* ^A^	583.65 ± 31.87* ^B^	521.71 ± 29.14* ^C^
Gain:feed ratio	LP	0.35 ± 0.01^a^	0.28 ± 0.01^ab^	0.20 ± 0.01^b^
	HP	0.42 ± 0.01* ^A^	0.34 ± 0.01* ^AB^	0.24 ± 0.01^B^

^1 ^Means ± SE without common letter differ significantly between body weight groups (lower case for LP group and upper case for HP).* indicating treatment differences at the same body weight (P < 0.05).

^2^LP, low protein diet.

^3^HP, high protein diet.

### Effect of dietary protein level on carcass traits

Dietary protein had no effect on carcass composition at 30 kg body weight, but affected the carcass composition significantly at 60 kg and 100 kg (table 4). Briefly, high dietary protein reduced the fat meat percentage and average backfat thickness (P < 0.05), but increased the lean meat percentage and loin eye area (P < 0.05). With increasing body weight, lean meat percentage diminished, whereas fat meat percentage, loin eye area and average backfat thickness increased.

**Table 4:** Effect of dietary protein on carcass composition^1^

		30 kg	60 kg	100 kg
Lean meat percentage(%)	LP	47.73 ± 1.01^a^	41.75 ± 0.77^b^	37.90 ± 2.13^c^
	HP	48.65 ± 1.02^A^	44.43 ± 2.13^B^	40.65 ± 3.35^C^
Fat meat percentage(%)	LP	20.82 ± 1.68^a^	34.10 ± 0.62^b^	39.17 ± 2.04* ^c^
	HP	18.68 ± 1.41^A^	31.53 ± 1.66^B^	36.00 ± 1.08^C^
Lean eye area (cm^2^)	LP	10.96 ± 0.12^a^	17.69 ± 1.52^b^	21.53 ± 1.66^c^
	HP	11.66 ± 1.24^A^	20.02 ± 1.59^B^	23.76 ± 1.22* ^C^
Average backfat thickness (cm)	LP	1.80 ± 0.13^a^	3.18 ± 0.20* ^a^	4.75 ± 0.16* ^a^
	HP	1.73 ± 0.10^A^	2.84 ± 0.26^B^	3.55 ± 0.14^C^

^1 ^Symbols/abbreviations as for table 3.

### Effect of dietary protein level on adipocyte size

**Figure 1:**
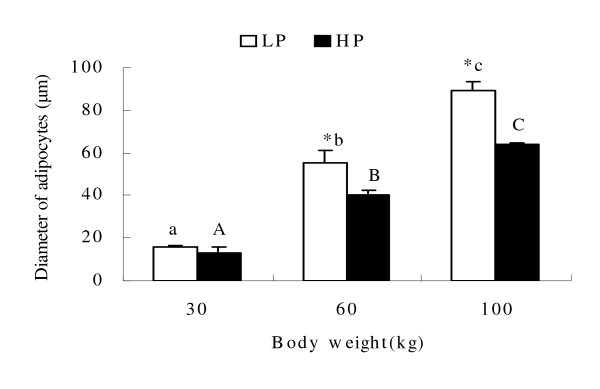
**Effect of dietary protein on adipocyte size**. Means ± SE without common letter differ significantly between body weight groups (lower case for LP group and upper case for HP) (P < 0.05). * indicates treatment differences at the same body weight (P < 0.05).

Figure 1 shows the effect of dietary protein on adipocyte size. High protein significantly reduced the adipocyte size at 60 and 100 kg (P < 0.05). Size in both groups increased with increasing body weight (P < 0.05).

### Effect of dietary protein level on serum parameters

Serum concentrations of FFA and TG were increased (P < 0.05) by high dietary protein at 60 and 100 kg body weights. Dietary protein had no effect on serum insulin and glucose concentrations at any body weight (table 5). FFA in both groups was highest at 60 kg compared with 30 kg and 100 kg. TG in both groups diminished with increasing body weight.

**Table 5:** Effect of dietary protein on serum parameters^1^

		30 kg	60 kg	100 kg
Free fatty acids (*μ*Eq/L)	LP	238.42 ± 12.33^a^	320.00 ± 17.11^b^	240.23 ± 11.32^c^
	HP	253.82 ± 11.22^A^	410.34 ± 16.22* ^B^	320.24 ± 12.42* ^C^
Triacylglycerol (mmol/L)	LP	0.38 ± 0.03^a^	0.47 ± 0.04^ab^	0.51 ± 0.02^b^
	HP	0.40 ± 0.02^A^	0.63 ± 0.07* ^AB^	0.75 ± 0.03* ^B^
Glucose (mg/dL)	LP	64.62 ± 3.77^a^	65.09 ± 2.11^a^	66.22 ± 5.76^a^
	HP	64.73 ± 4.01^A^	65.49 ± 1.76^A^	66.42 ± 2.99^A^
Insulin (*μ*IU/mL)	LP	6.76 ± 0.11^a^	7.99 ± 0.10^a^	7.52 ± 0.17^a^
	HP	6.63 ± 0.18^A^	7.49 ± 0.11^A^	7.22 ± 0.09^A^

^1 ^Symbols/abbreviations as for table 3

### Effect of dietary protein level on enzyme activities

High dietary protein significantly decreased the activities of ACC, FAS, ME and G-6-PDH at 60 and 100 kg, increased the activities of LPL and CPT-1 at 60 kg, but reduced activities of CPT-1 and LPL at 100 kg (P < 0.05) (table 6). However, HSL activity was decreased markedly at 60 kg but increased markedly at 100 kg. Lipid metabolism-related enzyme activities were not notably affected by dietary protein at 30 kg. The activities of ACC, FAS, ME and G-6-PDH enzymes in both LP and HP groups were up-regulated in all 3 weight groups, whereas activities of LPL, HSL and CPT-1 enzymes in both LP and HP groups at 60 kg were higher than at 30 kg and 100 kg.

**Table 6:** Effect of dietary protein level on adipose tissue enzyme activities^1^

		30 kg	60 kg	100 kg
ACC (nmol HCO_3_/min/mg protein)	LP	28.35 ± 1.12^a^	40.03 ± 2.00* ^ab^	47.43 ± 1.24* ^b^
	HP	24.12 ± 1.90^A^	26.04 ± 1.91^AB^	34.13 ± 1.25^B^
FAS (nmol NADPH/min/mg protein)	LP	33.41 ± 1.32^a^	69.03 ± 3.14* ^b^	72.23 ± 3.42* ^b^
	HP	35.34 ± 1.09^A^	54.04 ± 1.80^B^	60.41 ± 2.20^B^
ME (nmol NADPH/min/mg protein)	LP	253.43 ± 14.23^a^	290.33 ± 12.44* ^b^	320.34 ± 13.42* ^b^
	HP	234.12 ± 12.09^A^	240.43 ± 11.90^A^	253.11 ± 13.20^A^
G-6-PDH (nmol NADPH/min/mg protein)	LP	189.42 ± 12.34^a^	233.32 ± 17.43* ^b^	290.54 ± 14.67* ^c^
	HP	177.23 ± 12.12^A^	168.01 ± 18.65^A^	278.39 ± 15.77^B^
LPL (mmol oleic acid/h/g tissue)	LP	29.56 ± 1.45^a^	30.43 ± 1.22^a^	29.21 ± 1.88^a^
	HP	27.43 ± 1.34^A^	40.11 ± 1.13* ^B^	36.43 ± 1.01* ^B^
HSL (mU/mg protein)	LP	3.62 ± 0.16^a^	8.02 ± 0.08* ^b^	4.21 ± 0.06^a^
	HP	3.75 ± 0.13^A^	6.43 ± 0.02^B^	6.43 ± 0.06* ^B^
CPT-1 (nmol palmitate/min/mg protein)	LP	2.17 ± 0.09^a^	6.34 ± 0.11^b^	5.94 ± 0.10^b^
	HP	2.13 ± 0.05^A^	6.02 ± 0.09* ^B^	3.12 ± 0.06* ^A^

^1 ^Symbols/abbreviations as for table 3.

### Effect of dietary protein level on mRNA abundance of genes

**Figure 2:**
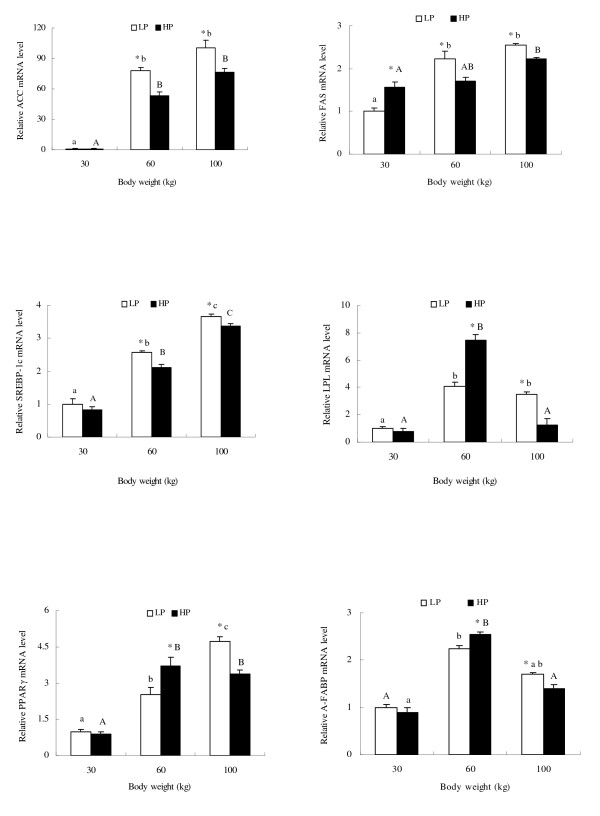
**Relative mRNA abundance of lipogenic genes in adipose tissues at 30, 60 and 100 kg body weights of pigs fed high and low dietary protein, based on extraction of total RNA and subsequent real-time PCR analysis**. Means ± SE without common letter differ significantly between body weight groups (lower case for LP group and upper case for HP). * indicates treatment differences at the same body weight (P < 0.05).

**Figure 3:**
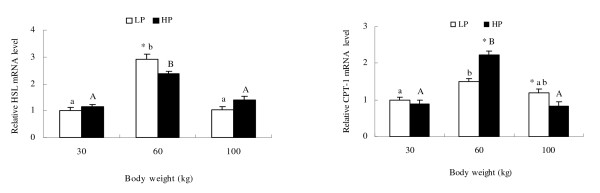
**Relative mRNA abundance of lipolysis genes in adipose tissues at 30, 60 and 100 kg body weights of pigs fed high and low dietary protein, based on extraction of total RNA and subsequent real-time PCR analysis**. Means ± SE without common letter differ significantly between body weight groups (lower case for LP group and upper case for HP). * indicates treatment differences at the same body weight (P < 0.05).

High dietary protein decreased levels of ACC, FAS and SREBP-1 mRNAs (P < 0.05) at 60 and 100 kg, promoted mRNA expression of LPL, CPT-1, PPAR *γ *and A-FABP at 60 kg (P < 0.05) and reduced mRNA expression of these genes at 100 kg (P < 0.05). HSL mRNA expression was reduced at 60 kg but was increased at 100 kg (figures 2 and 3). However, dietary protein did not significantly affect mRNA expression of these genes at 30 kg, with the exception that FAS mRNA level was increased by high dietary protein. The mRNA abundance of ACC, FAS, SREBP-1 and PPAR*γ *in LP and HP groups increased as weight increased from 30 kg to 100 kg, but mRNA abundance of LPL, A-FABP, HSL and CPT-1 in both groups was higher at 60 kg than at 30 kg and 100 kg.

### Effect of dietary protein level on protein expression level

**Figure 4:**
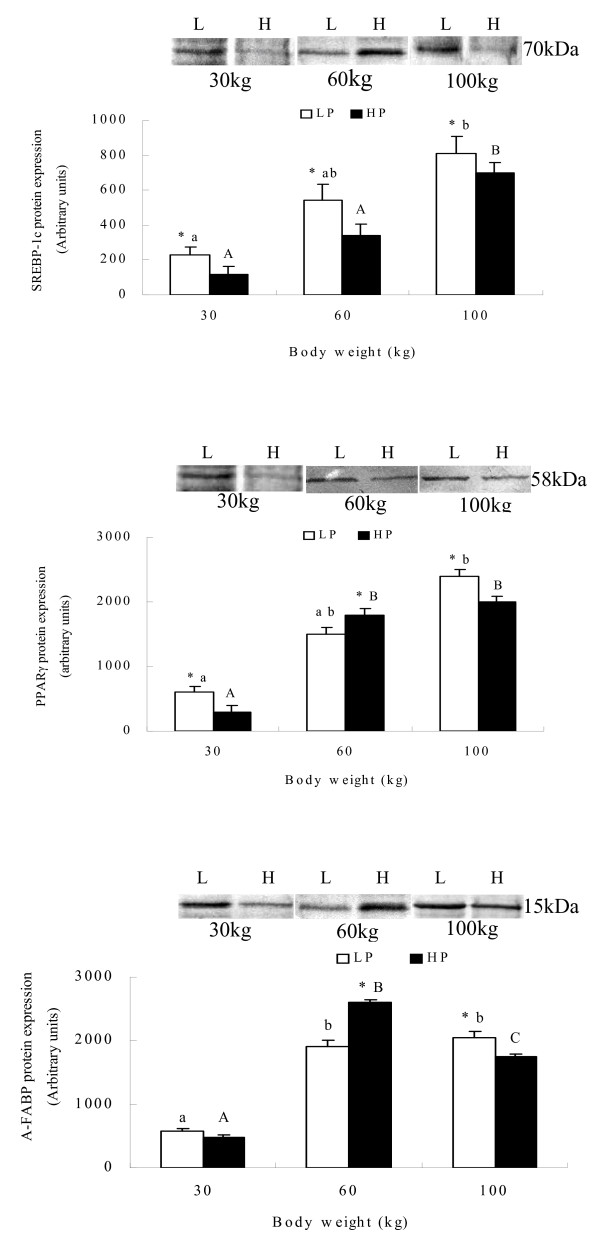
**Protein expressions in adipose tissues at 30, 60 and 100 kg body weights of pigs fed high and low dietary protein**. Means ± SE without common letter differ significantly between age groups (lower case for LP group and upper case for HP). * indicates treatment differences at the same body weight (P < 0.05).

High dietary protein significantly reduced SREBP-1 protein expression at 60 and 100 kg. Expression of PPAR*γ *and A-FABP was increased at 60 kg, but decreased at 100 kg (P < 0.05). The protein abundance of SREBP-1 and PPAR*γ *increased with increasing body weight. Dietary protein did not significantly affect the protein expression of these genes at 30 kg body weight (figure 4).

## Discussion

Pigs and humans share numerous physiological and phenotypic similarities with regard to fat deposition [20], and pigs have been used extensively as a biomedical research model for various human physiological conditions [21]. Obesity is the surplus fat deposition in the adipose tissue associated with nutrition [39]. Accumulation of fat may increase even during a period of decreased fat synthesis if there is simultaneously an even greater decrease in the turnover and catabolism of adipose tissue fatty acids. High dietary protein decreases fat deposition in adipose tissue [22-26,40-44]. However, there is little information on how dietary protein regulates the balance between synthesis, uptake, and transport of fatty acids. Therefore, in this study, we used the fatty Wujin pig as a model to investigate the regulating mechanisms of dietary protein on lipid metabolism in adipose tissue.

Previous studies on the market weight of swine have demonstrated that lower protein diets reduced [25,40] or had no effect [23,41] on weight gain and gain-to-feed ratio, but our observations were that higher dietary protein level increased the daily weight gain of Wujin pigs. This discrepancy may be due to the different porcine breeds used in the different studies. However, our results are the first report on the influence of dietary protein levels on growth performance of the Wujin pig, a typical fatty local Chinese breed of swine. Our data, therefore, provide the basis for studying differences in growth performance between Wujin pigs and other breeds of swine.

Increasing dietary protein has been well documented to decrease adipose tissue fat deposition and carcass backfat thickness in growing pigs [23-25,40,41], and our results are consistent with this. No differences in serum glucose and insulin were detected in Wujin pigs fed 14% versus 18% dietary protein in the present study, which is in agreement with reports from several other laboratories [45,46]. However, Caperna et al. [47] and Atinmo et al. [48] reported that feeding low protein diets to young pigs produced lower serum glucose and insulin levels than did high protein diets. High dietary protein has been reported to enhance serum FFA and TG contents in the late growing stage of pigs [49-51,38], an observation also made in the present study, especially at 60 kg and 100 kg body weights. Serum FFA results mainly from release from adipose tissue catalyzed by HSL or from hepatic triglycerides through the hydrolysis of LPL circulation. These data suggest that high dietary protein facilitates the release and transport of FFA from adipose tissue.

Mersmann et al. [52] reported a marked increase in adipocyte size accompanied by an increase in the size of the central lipid droplets with age. The present study showed that high protein reduced adipocyte size, with differences increasing with increasing body weight. Fat deposition is mainly associated with lipogenic capacity in adipose tissue. ACC is a key rate limiting enzyme in the *de novo *pathway of fatty acid synthesis [6]. The entire pathway of palmitate synthesis from malonyl-CoA is catalyzed by FAS which is a key determinant of the maximal capacity of a tissue to synthesize fatty acids by the *de novo *pathway [7,8]. ME and G-6-PDH are recognized to be the main enzymes involved in supplying NADH, the co-enzyme required for reductive biosynthesis of fatty acids [9-11]. SREBP-1, as a key transcription factor, regulates the expression of lipogenic genes such as FAS and ACC under different nutritional states, and is a candidate gene for determination of lipogenic capacity [12-14,53]. The present study showed that high dietary protein reduced the mRNA abundance or protein expression level of lipogenic genes such as ACC, ME, G-6-PDH and SREBP-1 during the whole growth period. Although FAS mRNA expression and enzyme activity were up-regulated at 30 kg by high dietary protein, the FAS gene was down-regulated at 60 and 100 kg.

Some reports have indicated that reductions in lipogenic activity are typically associated with reductions in activities of enzymes such as ACC, FAS and ME [6,8,38,54-58]. The down-regulated expression of these lipogenic genes means there is a reduced capacity for *de novo *synthesis of FFA. Additionally, our data show that gene mRNA expression paralleled enzyme activity and protein expression, in agreement with the report of Morris et al. [59]. Taken altogether, these data indicate that high dietary protein reduces the capacity of adipose tissue for *de novo *synthesis of FFA, leading to decreasing fat accumulation.

Fat deposition is determined by a complex balance between lipogenic, lipolytic enzymes and fatty acids transport, as well as fatty acid utilization. LPL is the major enzyme responsible for hydrolysis of triglycerides in chylomicrons and VLDL to provide free fatty acids for tissue utilization or storage [12]. LPL is abundant in adipose tissue, and its gene expression correlates strongly with the uptake of lipid fuels by the tissue. Therefore, LPL is considered the key factor determining lipid deposition in tissues, and has been described as the 'metabolic gatekeeper' [60-62]. Our results show that high dietary protein increased LPL mRNA expression and enzyme activity at 60 kg, which suggests that more FFA was released from VLDL at this weight. This situation was reversed at 100 kg body weight.

PPAR*γ *is a member of the nuclear receptor superfamily and regulates the expression of several genes encoding proteins involved in adipocyte differentiation and fat deposition [17]. It has been reported to promote LPL and FABP gene expression and to suppress the HSL gene [17]. HSL cleaves fatty acids from intracellular triacylglycerol for oxidation and export [12]. CPT-1 initiates mitochondrial import in the degradation of fatty acids through mitochondrial *β*-oxidation [14]. The present study has shown that PPAR*γ*, A-FABP and CPT-1 and mRNA and protein expression were up-regulated by high dietary protein at 60 kg and down-regulated at 100 kg, with the opposite occurring with HSL. This suggests that high dietary protein decreases the release of FFA from adipose tissue at 60 kg. FFA bind to FABP and are transported for esterification and oxidation in adipose tissue [18]. Therefore, high dietary protein regulates FFA uptake, transport and lipolysis differently at different growth stages.

The rate of lipogenesis in adipose tissue has been found to decrease during aging [63,64]. This decreased lipogenesis accompanying maturity is of particular interest because it has been noted for many years that fat deposition increases with age [65]. Thus, an apparent contradiction exists: fat accumulation is increasing when the rate of lipogenesis is decreasing. This may be due to the balance between synthesis and degradation. A net increase in the accumulation of fat could result even during a period of decreased fat synthesis if there is simultaneously an even greater decrease in the turnover and catabolism of adipose tissue fatty acids [66]. However, some reports indicate that lipogenic activity is greatest at 4 months of age in pig adipose tissue [53,67]. Other reports have shown that lipogenic enzyme activities generally decreased with increasing weight in pigs [57,52,68].

The above studies on fat deposition in porcine adipose tissue have focused mainly on lipogenesis, and there is little information on lipolysis. Our study is the first to investigate in detail the developmental changes in lipid metabolism-related gene expression and enzyme activity controlled by lipogenic and lipolytic genes throughout the growth-finishing stages. Our results have shown that the enzyme activities/protein expression levels and gene expression of ME, ICD, G-6-PDH, ACC, FAS and SREBP-1 increased with increasing body weight. However, mRNA and protein expression of HSL, LPL, A-FABP and CPT-1 activity peaked at 60 kg, indicating that different lipid metabolic activities exist at different body weights.

## Conclusion

**Figure 5:**
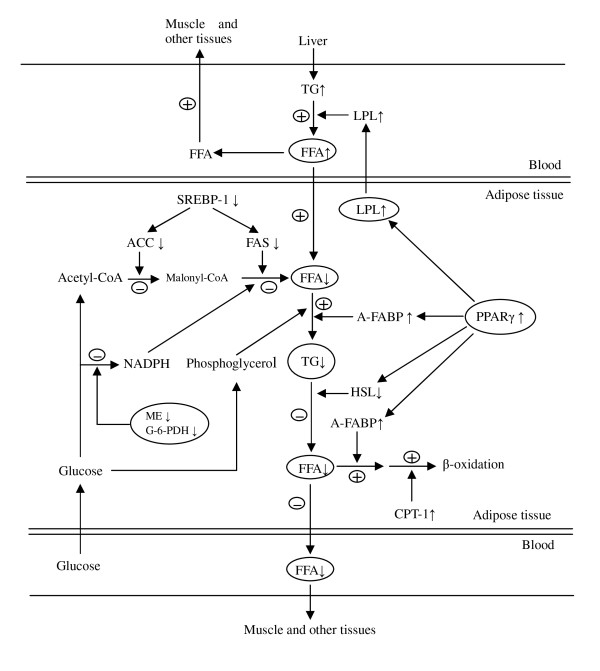
**Impact of high dietary protein on lipid metabolism in porcine adipose tissue at body weight 60 kg**. ↑: up-regulation of gene expression or increased concentration of metabolite. ↓: down-regulation of gene expression or enzyme activity or decreased concentration of metabolite. +: enhanced metabolic pathway; -: diminished metabolic pathway.

**Figure 6:**
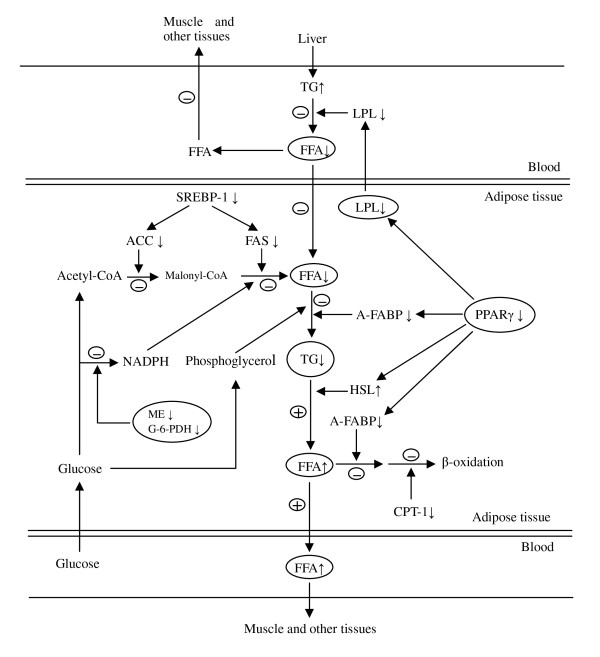
**Impact of high dietary protein on lipid metabolism in porcine adipose tissue at body weight 100 kg**. ↑: up-regulation of gene expression or increased concentration of metabolites. ↓: down-regulation of gene expression or enzyme activity or decreased concentration of metabolite. +: enhanced metabolic pathway; -: diminished metabolic pathway. At body weight 30 kg, there were no differences except that gene expression of FAS was up-regulated.

In all, our observations have indicated that the mechanism of down-regulation of synthesis of triglyceride in adipose tissue by high dietary protein is mainly by inhibition of lipogenic gene expression. It appears that high dietary protein regulates FFA uptake, transport and lipolysis differently at different growth stages. High dietary protein promoted FFA uptake and oxidation in adipose tissue at 60 kg, but increased FFA release from adipose tissue to serum, and decreased FFA oxidation in adipose tissue at 30 and 100 kg (figures 5 and 6).

## Abbreviations

RT-PCR: reverse transcription and polymerase chain reaction; SREBP-1c: sterol regulatory element binding protein 1c; FAS: fatty acid synthase; ACC: acetyl-CoA carboxylase; A-FABP: Adipocyte (A) - fatty acid binding proteins; CPT-1B: carmitine palmtoyltransferase-1B; HSL: hormone sensitive lipase; LPL: lipoprotein lipase; ME: malic enzyme; G-6-PDH: glucose-6-phosphate dehydrogenase; PPAR*γ*: peroxisome proliferator-activated receptor *γ*; FFA: free fatty acid(s); TG: triacylglycerol.

## Competing interests

The authors declare that they have no competing interests.

## Authors' contributions

ZSM analyzed the data and wrote this manuscript. WJ and SXL carried out the experiments. ZX and GCR participated in the experimental design. GSZ designed the experiments and edited the manuscript. All authors read and approved the final manuscript.
